# New Books

**Published:** 2004-08

**Bioethics in Complexity: Foundations and Evolutions**

Sergio De Risio, Franco F Orsucci, eds.

London:Imperial College Press, 2004. 100 pp. ISBN: 1-86094-399-3, $34

**Bioinformatics, Biocomputing and Perl: An Introduction to Bioinformatics Computing Skills and Practice**

Michael Moorhouse, Paul Barry

Hoboken, NJ:John Wiley & Sons, Inc., 2004. 506 pp. ISBN: 0-470-85331-X, $65

**Bioinformatics: Sequence and Genome Analysis**

David Mount, David W. Mount

Plainview, NY:Cold Spring Harbor Laboratory Press, 2004. 600 pp. ISBN: 0-8796-9687-7, $150

**Computational Genomics: Theory and Application**

Richard P. Grant, ed.

Norwich, UK:Horizon Scientific Press, 2004. 306 pp. ISBN: 1-904933-01-7, $190

**Dictionary of Bioinformatics and Computational Biology**

John M. Hancock, Marketa J. Zvelebil, eds.

Hoboken, NJ:John Wiley & Sons, Inc., 2004. 664 pp. ISBN: 0-471-43622-4, $99.95

**Digital Code of Life: How Bioinformatics Is Revolutionizing Science, Medicine, and Business**

Glyn Moody

Hoboken, NJ:John Wiley & Sons, Inc., 2004. E-book, ISBN: 0-471-68964-5, $34.95

**DNA Amplification: Current Technologies and Applications**

Vadim V. Demidov, Natalia E. Broude

Norwich, UK:Horizon Scientific Press, 2004. 336 pp. ISBN: 0-9545232-9-6, $180

**Encyclopedia of Medical Genomics and Proteomics**

Jurgen Fuchs, Mauriziio Podda

New York:Marcel Dekker, Inc., 2004. 1200 pp. ISBN: 0-8247-4794-1, $420

**Genetic Databases: Socio-Ethical Issues in the Collection and Use of DNA**

Oonagh Corrigan; Richard Tutton

New York:Routledge, 2004. 224 pp. ISBN: 0415316804, $36.95

**Genomics and Proteomics in Nutrition**

Carolyn D. Berdanier, Naima Moustaid-Moussa, eds.

New York:Marcel Dekker, Inc., 2004. 500 pp. ISBN: 0-8247-5430-1, $175

**How the Human Genome Works**

Edwin H. McConkey

Sudbury, MA:Jones and Bartlett Publishers, Inc., 2004. 118 pp. ISBN: 0-7637-2384-3, $24.95

**Molecular Toxicology Protocols**

Phouthone Keohavong, Stephen G. Grant

Totowa, NJ:Humana Press, 2004. 550 pp. ISBN: 1-58829-084-0, $125

**Practical Bioinformatics**

Janusz M. Bujnicki

New York:Springer-Verlag, Inc., 2004. 265 pp. ISBN: 3-540-20613-2, $189

**Protein Bioinformatics: An Algorithmic Approach to Sequence and Structure Analysis**

Ingvar Eidhammer, Inge Jonassen, William R. Taylor

Hoboken, NJ:John Wiley & Sons, Inc., 2004. 376 pp. ISBN: 0-470-84839-1, $85

**Protein Expression Technologies: Current Status and Future Trends**

François Baneyx

Norwich, UK:Horizon Scientific Press, 2004. 532 pp. ISBN: 0-9545232-5-3, $180

**Proteome Analysis: Intrepreting the Genome**

David Speicher

Burlington, MA:Elsevier Science & Technology, 2004. 400 pp. ISBN: 0-444-51024-9, $165

**The Hope, Hype, and Reality of Genetic Engineering**

John C. Avise

New York:Oxford University Press, 2004. 256 pp. ISBN: 0-19-516950-6, $35

**The Stored Tissue Issue: Biomedical Research, Ethics, and Law in the Era of Genomic Medicine**

Robert F. Weir, Robert S. Olick, Jeffrey C. Murray

New York:Oxford University Press, 2004. 368 pp. ISBN: 0-19-512368-9, $46.50

**Chemical Genomics**

Ferenc Darvas, Andras Guttman, Gyorgy Dorman

New York:Marcel Dekker, Inc., 2004. 280 pp. ISBN: 0-8247-5490-5, $135

